# Retraction: Determination and production of antimicrobial compounds by *Aspergillus clavatonanicus* strain MJ31, an endophytic fungus from *Mirabilis jalapa L*. using UPLC-ESI-MS/MS and TD-GC-MS analysis

**DOI:** 10.1371/journal.pone.0268371

**Published:** 2022-05-26

**Authors:** The PLOS ONE Editors

Following the publication of this article [1], concerns were raised that the mass spectrometry spectra presented in Fig 8 appear similar to the mass spectrometry spectra presented in Fig 6 of [2], Fig 3 of [3 retracted in 4], and Fig 4 of [5]. Furthermore, concerns were raised that the discovery of an *Aspergillus* species capable of producing synthetic and semi-synthetic antifungals and antibiotics requires a higher burden of proof than is demonstrated in this article [1]. Following these concerns the article was reassessed by two members of the *PLOS ONE* Editorial Board, who raised the following additional concerns:

1. The study lacks appropriate controls to rule out sample contamination, including contamination of the UPLC system, the C18 column, or the culture extract.
2. The UPLC and MRM-extracted ion chromatograms of the extracts compared to the standard have not been reported in the article. Although the study reports such data obtained on the standards, the data obtained on the extracts or the curcumin and palamatine controls are missing from the article. The editorial board members commented that the study is not reproducible in the absence of these data, and the reliability of the analysis cannot be assessed.
3. The description of extracts preparation is inadequate, and there is no indication as to the yield of dried extract obtained per culture or mycelial mass weight.
4. The data presented in the study were obtained from a single extract, as opposed to multiple independently prepared fungal extracts as would be required according to appropriate scientific methodology. In the absence of extractions obtained from repeat experiments, contamination of the single extract used for this study cannot be ruled out. The editorial board members commented that lack of analysis of samples obtained from at least three independent cultures is a major shortcoming of the work reported in this study.
5. Figs 4 and 5, as well as Table 1, do not include appropriate controls. For Fig 4, test organisms grown separately are required to demonstrate uninhibited growth. In Fig 5 and Table 1 the MICs/sensitivity of these organisms as well as the *Aspergillus* strain to the antifungals found to be produced by the *Aspergillus* strain should be presented.

The corresponding author explained that the spectra of the standards presented in this article [1] and related articles [2, 3 retracted in 4, 5] are the same spectra, as the extracts tested in these studies were sent for analysis at the CSIR-CDRI Lucknow for detection of compounds, and the standards as well as the extracts reported in these studies were run at the same time.

Regarding point 1, the corresponding author provided data on the blank controls run between samples to rule out contamination of the UPLC system or antimicrobial compound bleeding from the column. The board members commented that the authors adequately addressed the concerns regarding potential UPLC system or column contamination, but that the information provided was insufficient to rule out contamination of the culture extract.

Regarding point 3, the corresponding author provided details regarding the weight of ethyl acetate mass extracted, but indicated that they are unable to report on the yield as the mass of dried mycelia was not measured before extraction. The editorial board members comment that the yield value is required to be able to estimate the concentration of antibiotics present in the culture, and the yield would give an indication of whether the levels found are consistent with potential contamination from laboratory settings where concentrated antibiotic and antifungal stocks are commonly used. Ruling out potential contamination is particularly important considering all the antibiotic and antifungal compounds found in the extract were used at some point in the different assays described in the article.

Regarding point 5, the corresponding author clarified that test organisms were grown separately to demonstrate uninhibited growth, and provided an updated Fig 4 presenting these additional controls. The corresponding author also provided several references to support the MICs for miconazole against *Fusarium* species, but they did not provide data that confirm the MICs reported in these studies. However, the board members state that the testing and reporting of MICs for the strains used in Fig 5 and Table 1 are essential to the article so as to demonstrate that the *Aspergillus* strain reportedly producing the fungicides is resistant to the concentrations of the compounds, at least at the levels found in the media.

Individual level data underlying most results presented in this study were submitted to the journal, but they were not sufficient to resolve the concerns pertaining to the study design.

In light of the unresolved concerns listed in points 1–5 above, that question the validity and reliability of the data presented in this article, the *PLOS ONE* Editors retract this article.

The standards for fluconazole, chloramphenicol, rifampicin, and streptomycin in Fig 8 reported in this article [1] have previously been reported in [2] which is not offered under a CC-BY license. These results presented in Fig 8 are therefore not offered under the Creative Commons Attribution Licence. In addition, the spectra for miconazole and ketoconazole were previously reported in [3 retracted in 4, and 5], which have not been cited appropriately. At the time of retraction, the article [1] was republished to update the copyright statement, the figure legend of Fig 8, and the reference list accordingly.

BPS, VKM, AKP, VVL, SU, and VKG disagree with the retraction and stand by the article’s findings. PC, BK, and ST either did not respond directly or could not be reached.

## References

[1] Mishra VK, Passari AK, Chandra P, Leo VV, Kumar B, Uthandi S, et al. (2017) Determination and production of antimicrobial compounds by *Aspergillus clavatonanicus* strain MJ31, an endophytic fungus from *Mirabilis jalapa L*. using UPLC-ESI-MS/MS and TD-GC-MS analysis. PLoS ONE 12(10): e0186234. 10.1371/journal.pone.0186234 29049321 PMC5648158

[2] Passari AK, Chandra P, Zothanpuia, Mishra VK, Leo VV, Gupta VK, et al. (2016) Detection of biosynthetic gene and phytohormone production by endophytic actinobacteria associated with *Solanum lycopersicum* and their plant-growth-promoting effect. Res. Microbiol. 167(8). 10.1016/j.resmic.2016.07.001 27421813

[3] Zothanpuia Passari AK, Chandra P Leo VV, Mishra VK, Kumar Band Singh BP (2017) Production of Potent Antimicrobial Compounds from *Streptomyces cyaneofuscatus* Associated with Fresh Water Sediment. Front. Microbiol. 8:68. doi: 10.3389/fmicb.2017.00068 28179900 PMC5263160

[4] Editorial Office Frontiers (2018) Retraction: Production of Potent Antimicrobial Compounds from *Streptomyces cyaneofuscatus* Associated with Fresh Water Sediment. Front. Microbiol. 9. doi: 10.3389/fmicb.2018.01681 30034387 PMC6049523

[5] Passari AK, Mishra VK, Singh G, Singh P, Kumar B, Gupta VK, et al. (2017) Insights into the functionality of endophytic actinobacteria with a focus on their biosynthetic potential and secondary metabolites production. Sci Rep 7, 11809. 10.1038/s41598-017-12235-4 28924162 PMC5603540

